# Abnormal Glucose Metabolism and Insulin Resistance Are Induced via the IRE1α/XBP-1 Pathway in Subclinical Hypothyroidism

**DOI:** 10.3389/fendo.2019.00303

**Published:** 2019-05-17

**Authors:** Chao Xu, Lingyan Zhou, Kunpeng Wu, Yujie Li, Jin Xu, Dongqing Jiang, Ling Gao

**Affiliations:** ^1^Department of Endocrinology and Metabolism, Shandong Provincial Hospital affiliated to Shandong University, Jinan, China; ^2^Institute of Endocrinology, Shandong Academy of Clinical Medicine, Jinan, China; ^3^Shandong Provincial Key Laboratory of Endocrinology and Lipid Metabolism, Jinan, China; ^4^Department of Endocrinology and Metabolism, The Second Hospital of Shandong University, Jinan, China; ^5^Department of Hematology, Huashan Hospital, Fudan University, Shanghai, China; ^6^Scientific Center, Shandong Provincial Hospital affiliated to Shandong University, Jinan, China

**Keywords:** glucose impairment, Subclinical hypothyroidism, insulin resistance, ER stress, IRE1α/XBP-1

## Abstract

Subclinical hypothyroidism (SCH) and diabetes mellitus are closely related and often occur together in individuals. However, the underlying mechanism of this association is still uncertain. In this study we re-analyzed the data of a mature database (NHANES, 1999 ~ 2002) and found that both fasting plasma glucose levels and the proportion of hyperglycemic subjects among SCH patients were higher than that found in euthyroid controls. SCH was also associated with a 2.29-fold increased risk for diabetes. Subsequently, we established an SCH mouse model and subjected it to an oral glucose tolerance test (OGTT) and an insulin tolerance test (ITT). SCH mice exhibited impaired glucose and insulin tolerance. Increased HOMA-IR and decreased ISI indexes, indicating insulin resistance (IR), were also observed in the SCH state. Hepatic ERp29 and Bip, as well as IRE1α and XBP-1s, were induced significantly in SCH mice, suggesting the induction of endoplasmic reticulum (ER) stress, particularly involving the IRE1α/XBP-1s pathway. Interestingly, when we relieved ER stress using 4-phenyl butyric acid, abnormal glucose metabolism, and IR status in SCH mice were improved. Our findings suggest that ER stress, predominantly involving the IRE1α/XBP-1s pathway, may play a pivotal role in abnormal glucose metabolism and IR in SCH that may help develop potential strategies for the prevention and treatment of diabetes.

## Introduction

Subclinical hypothyroidism (SCH), characterized by elevated serum thyroid stimulating hormone (TSH) and normal serum free thyroxine (FT4) levels, is an independent risk factor for atherosclerosis, myocardial infarction, and non-alcoholic fatty liver disease (NAFLD) (1–4). Notably, emerging evidence shows that SCH and Type 2 diabetes mellitus (T2DM) are closely related and often occur together in individuals (5). We wondered whether SCH is also an independent risk factor for T2DM. The answer is still inconsistent. Epidemiological and clinical evidence indicates that SCH is positively correlated with insulin levels, HOMA-IR (Homeostasis Model Assessment-Insulin Resistance) values and diabetes (6–10). However, there have been some inconsistent findings that showed no significant difference between SCH and the euthyroid state (11, 12). Therefore, it is considered necessary to further investigate the relationship between SCH and glucose metabolism as this has not been fully elucidated.

Accumulating data suggest that endoplasmic reticulum (ER) stress is triggered in SCH. And ER stress is known to play an important role in the development of diabetes, metabolic syndrome and NAFLD by aggravating insulin resistance (IR), oxidative stress, inflammation, apoptosis, and other factors. Our previous findings also revealed that ER stress may play a pivotal role in lipid metabolic disorders in SCH (13). However, currently, there are no literature reports correlating ER stress with abnormal glucose metabolism and IR in SCH.

In this study, we first assessed glucose metabolism in SCH patients. We then used an SCH mouse model to mimic the SCH phenotype in humans and elucidate the influence of SCH on glucose metabolism and IR. Subsequently, we explored the role and underlying mechanism of ER stress in modulating glucose metabolism and IR in SCH. Our findings may provide greater understanding of the pathophysiological process of glucose metabolism and IR in SCH and indicate new intervention targets for the prevention and treatment of diabetes.

## Results

### Abnormal Glucose Metabolism in SCH Patients

In order to understand the features of glucose metabolism in SCH patients, we re-analyzed the data of the National Health and Nutrition Examination Survey (NHANES) from 1999 to 2002 (14). The selection process for patients to be included in this study is presented in Supplementary Figure 1.

A total of 1,318 respondents were included in this study; 54 (4.1%) were found to fit the criteria defined for SCH. The demographic characteristics of the SCH patients and euthyroid controls are presented in Table 1. We found that SCH was more common in non-Hispanic whites and in the elderly, which is consistent with a previous report (15). In the SCH group, the number of women was approximately twice that of men. However, there was no significant difference (*p* = 0.063), possibly due to the small sample size.

**Table 1:** Demographics of the sample population.

**Characteristic**	**Subclincal Hypothyroidism (*n* = 54)**	**Control (*n* = 1,264)**	***p*-value**
Sex			0.063
Male. No. (%)	19 (35.2%)	608 (48.1%)	
Female. No. (%)	35 (64.8%)	656 (51.9%)	
Race			0.049
Non-Hispanic white. No. (%)	32 (59.3)	584 (46.2)	
Black. No. (%)	4 (7.4)	262 (20.7)	
Mexican-American. No. (%)	14 (25.9)	309 (24.4)	
Other No. (%)	4 (7.4)	109 (8.6)	
Age			< 0.001
Youth (18–44y) No. (%)	12 (22.2)	650 (51.4)	
Middle age (45–65y) No. (%)	19 (35.2)	372 (29.4)	
Elderly (>65y) No. (%)	23 (42.6)	242 (19.1)	

**Figure 1:**
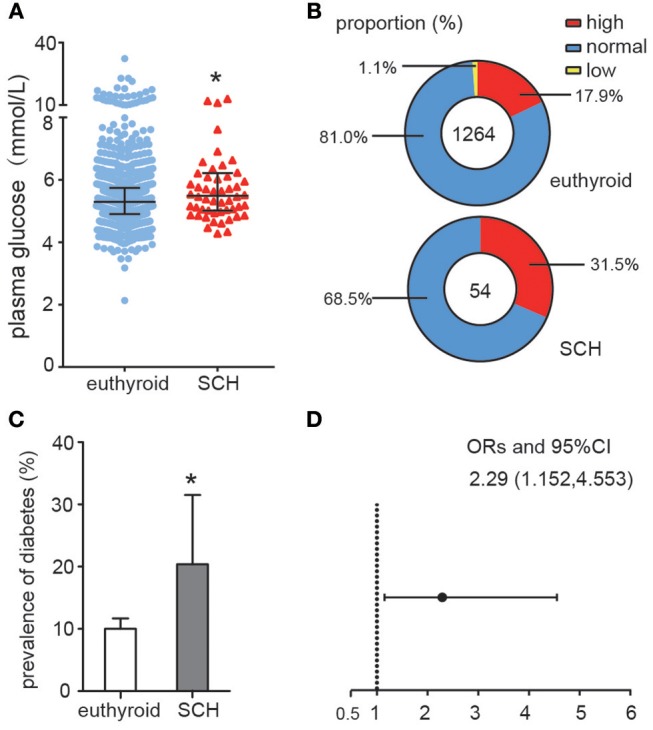
Abnormal Glucose metabolism in SCH patients. **(A)** Plasma glucose distributions in different group (euthyroid controls and SCH patients), data were expressed as: median (P25, P75). **(B)** The proportion of the glucose categories in different group. **(C)** The prevalence of diabetes in different group. The error bars represent the 95% CI of the ratio. **(D)** The odds ratio of diabetes in different group.

As shown in Figure 1A, plasma glucose levels in SCH patients were significantly higher than those in euthyroid controls as determined by the Mann-Whitney *U*-test analysis (*z* = −2.008, *p* = 0.045). When the glucose values were subdivided into different categories: high (GLU > 6.0 mmol/L), normal (3.9 mmol/L ≤ GLU ≤ 6.0 mmol/L) and low (GLU < 3.9 mmol/L), the proportion of individuals with high glucose level in the SCH group was clearly much higher than that in the euthyroid group (*p* = 0.038) (Figure 1B). Similarly, the prevalence of diabetes was nearly double that in the SCH group compared to that in the euthyroid group (Figure 1C). Using logistic regression, we found that the risk for diabetes increased 2.29-fold among subjects with SCH (Figure 1D). These data indicated that SCH patients are more prone to glucose metabolism disorders and have higher risk of diabetes.

### SCH Mice Exhibited Abnormal Glucose Metabolism and IR

In order to explore the mechanism of impaired glucose metabolism and IR in SCH, we first established an SCH mouse model by administering methimazole (0.08 mg/kg·BW·d, MMI) to the mice in their drinking water for 12 weeks (Supplementary Figure 2). In comparison with controls, SCH mice presented increased TSH and normal FT4 levels. The SCH state was successfully maintained from the 12th week to the 20th week after treatment with MMI, as demonstrated by us previously (13).

**Figure 2:**
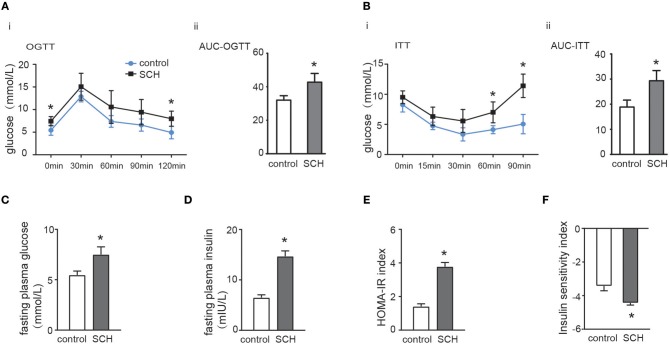
SCH mice exhibited abnormal glucose metabolism and IR. Male C57/BL6 mice were given methimazole (MMI, 0.08 mg/kg·BW·d, SCH group) or a corresponding volume of vehicle (control group). **(A)** Oral glucose tolerance test (OGTT) was implemented to detect glucose tolerance at the 13th week of administering MMI to mice in the drinking water. **(B)** Insulin tolerance test (ITT) was implemented to assess insulin-stimulated glucose disposal at the 14th week of administering MMI to mice in the drinking water. **(C)** The fasting plasma glucose level was assayed at the 14th week (*n* = 4–6). **(D)** The fasting plasma insulin level was assayed at the 14th week (*n* = 4–6). **(E)** Homeostasis Model Assessment-Insulin Resistance (HOMA-IR) index was calculated. **(F)** Insulin sensitivity index (ISI) was calculated. The data are presented as the mean ± SD. ^*^p < 0.05 compared with control.

Oral glucose tolerance testing was carried out at the 13th week and insulin tolerance testing at the 14th week to evaluate glucose metabolism and IR status. As shown in Figure 2A, both fasting glucose and postprandial glucose (120 min) levels were significantly higher in SCH mice than in controls. The area under the OGTT curves (AUC) consistently indicated that SCH mice had clearly impaired glucose tolerance. In the case of the ITT (Figure 2B), although both groups reached the lowest plasma glucose level at 30 min, glucose levels at 60 and 90 min were more elevated in SCH mice than in controls after injection of the same dose of insulin (*p* < 0.05). Meanwhile, both fasting glucose and insulin levels in SCH mice increased significantly at the 14th week (*p* < 0.05, Figures 2C,D). The Homeostasis Model Assessment-Insulin Resistance (HOMA-IR) index is widely used as an indicator of fasting IR while the insulin sensitivity index (ISI) is an indicator of insulin-sensitivity. In accordance with the increased fasting glucose and insulin levels, SCH mice presented higher HOMA-IR indexes, and lower ISI indexes compared to control mice (*p* < 0.05, Figures 2E,F). These results confirmed that abnormal glucose metabolism and clear IR were induced in SCH mice.

### Hepatic ER Stress Was Triggered in SCH Mice

Impaired glucose metabolism and IR were reported to be closely correlated with ER stress (16) which could cause ectopic phosphorylation of insulin receptor substrate (IRS) and impair insulin signal conduction (17). However, it was still unclear whether ER stress is also involved in IR found in SCH. Therefore, we first determined whether hepatic ER stress was induced in SCH mice.

**Figure 3:**
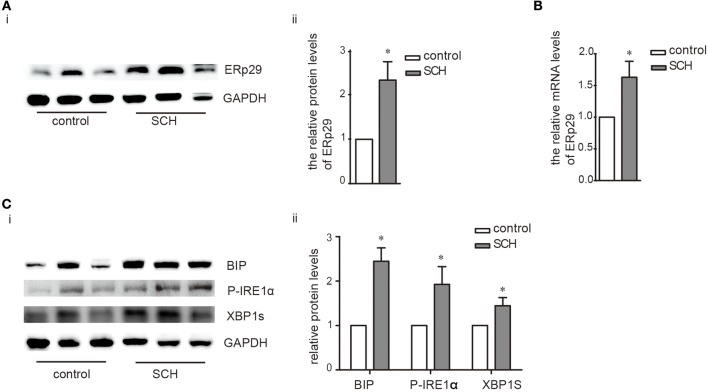
Hepatic ER stress was induced in SCH mice. Male C57/BL6 mice were given methimazole (MMI, 0.08 mg/kg·BW·d, SCH group) or a corresponding volume of vehicle (control group) for 14 weeks, the expression of proteins in ERS pathway were detected by western blot (*n* = 4–6). **(A)** The protein expression of ERq29 was detected by western blot. **(B)** The mRNA expression of ERq29 was detected by Real-time PCR. **(C)** The expression of proteins in the Bip/IRE1α/XBP-1 pathway were detected by western blot. The data are presented as the mean ± SD. ^*^p < 0.05 compared with control.

ER resident 29 kDa protein (ERp29) is one of the highly conserved ER chaperone proteins which is activated in response to ER stress (18). Compared to the controls, ERp29 mRNA expression, and protein synthesis were induced significantly in the liver of SCH mice (Figures 3A,B). The expression of binding immunoglobulin protein (Bip), a major ER luminal protein, was also induced significantly (Figure 3C). At the same time, SCH mice presented increased the expression of p-JNK/t-JNK in comparison with controls, indicating the activation of JNK (Supplementary Figure 3). Interestingly, expression of p-IRE1α (the active form of IRE1α) and XBP-1s (an active spliced form of XBP-1) in SCH mice was also markedly upregulated (Figure 3C). In our previous study, we demonstrated that the two other alternative pathways of ER stress (PERK/eif2α and ATF6) were not affected in SCH mice (13). Therefore, we did not measure the expression of these molecules. Based on these results, we concluded that hepatic ER stress was triggered in the SCH state and the IRE1α/XBP-1 pathway was activated.

### ER Stress May Play a Critical Role in Abnormal Glucose Metabolism and IR Induced by SCH

**Figure 4:**
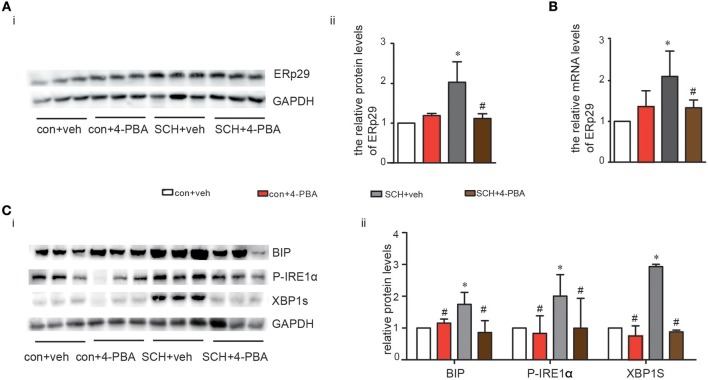
4-PBA alleviated hepatic ERS in SCH mice. We established SCH mouse model using MMI. When MMI was applied for 14 weeks, 4-PBA was given at a dose of 100 mg/kg·BW·d for 4 weeks, and mice were divided into four subgroups (as shown in Supplementary Figure 1): vehicle treated control mice group (con + veh group), 4-PBA treated control mice group (con + 4-PBA group), vehicle treated SCH mice group (SCH + vehicle group), and 4-PBA treated SCH mice group (SCH + 4-PBA group). **(A)** The protein expression of ERq29 was detected by western blot (*n* = 4–6). **(B)** The mRNA expression of ERq29 was detected by Real-time PCR. **(C)** The expression of proteins in the Bip/IRE1α/XBP-1 pathway were detected by western blot (*n* = 4–6). The data are presented as the mean ± SD. ^*^p < 0.05 compared with control. ^#^*p* < 0.05 compared with SCH.

4-phenylbutyrate (4-PBA, a chemical chaperone that can inhibit ER stress in cells) can improve ER folding capacity and facilitate the trafficking of mutant proteins by stabilizing their conformation (19). Consequently, we treated mice with 4-PBA for 4 weeks to alleviate ER stress. Notably, TSH and FT4 levels were not affected by the administration of 4-PBA (Supplementary Figure 4). As shown in Figures 4A,B, both the protein and mRNA expression of ERp29 decreased in SCH mice after 4-BPA treatment. The expression of Bip, p-IRE1α, and XBP-1s in 4-PBA-treated SCH mice was clearly and consistently down-regulated compared to those in the vehicle-treated SCH mice (Figure 4C). These data demonstrated that hepatic ER stress in SCH mice is ameliorated by 4-PBA.

**Figure 5:**
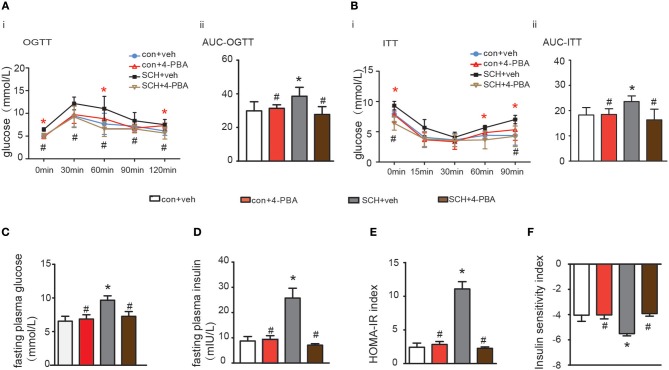
4-PBA alleviated abnormal glucose metabolism and IR in SCH mice. **(A)** Oral glucose tolerance test (OGTT) was implemented to detect glucose tolerance at the 17th week of administering MMI to mice in the drinking water (*n* = 4–6). **(B)** Insulin tolerance test (ITT) was implemented to assess insulin-stimulated glucose disposal at the 18th week of administering MMI to mice in the drinking water (*n* = 4–6). **(C)** The fasting plasma glucose level was assayed at the 18th week (*n* = 3–4). **(D)** The fasting plasma insulin level was assayed at the 18th week (*n* = 3–4). **(E)** Homeostasis Model Assessment-Insulin Resistance (HOMA-IR) index was calculated. **(F)** Insulin sensitivity index (ISI) was calculated. The data are presented as the mean ± SD. ^*^p < 0.05 compared with vehicle treated mice group. #*p* < 0.05 compared with 4-PBA treated SCH mice group.

Glucose metabolism in the 4-PBA-treated SCH mice was assessed and as shown in Figures 5A,B, impaired glucose tolerance detected by OGTT was strongly alleviated after 4-PBA treatment for 4 weeks. Insulin sensitivity, as indicated by ITT, in the 4-PBA-treated SCH group was also improved. Serum fasting glucose and insulin levels in the 4-PBA-treated SCH mice decreased into the normal range following inhibition of ER stress (Figures 5C,D). Treatment with 4-PBA also ameliorated both HOMA-IR and ISI indexes in SCH mice (Figures 5E,F). Thus, inhibiting ER stress using 4-PBA in mice significantly alleviated SCH-induced abnormal glucose metabolism and IR. Together, these findings confirmed that hepatic ER stress play an important role in abnormal glucose metabolism and IR associated with SCH.

## Discussion

Our findings demonstrated that hepatic ER stress is induced in abnormal glucose metabolism and phenotypic IR in SCH mice. In addition, the IRE1α/XBP-1 pathway was predominately involved in the process. The amelioration of ER stress by 4-PBA suggests a useful strategy for the alleviation of abnormal glucose metabolism and IR in SCH.

SCH, the most common thyroid dysfunction, is closely associated with IR (20), A recent meta-analysis analyzed nine studies (including seven cross-sectional and two case-control studies) and concluded that SCH was significantly associated with a higher risk of metabolism syndrome, in which the pathogenic basis is IR (21). Notably, although not entirely consistently (11), a large number of studies have demonstrated that serum TSH concentrations in SCH patients were positively associated with HOMA-IR index or insulin levels (6, 22, 23). In this study, we first re-analyzed the epidemiological data from NHANES which provided an invaluable database unmatched by any other in size or content. In agreement with previous studies, we found that both fasting plasma glucose levels and the proportion of hyperglycemic subjects among SCH patients were higher than in euthyroid controls. SCH was also associated with a 2.29-fold increased risk for diabetes. Using a proven and well-established database provides more reliable information and improves the power of derived statistics. Subsequently, we established an SCH mice model to simulate the SCH phenotype observed in humans. Consistently, SCH mice consistently exhibited prominent hyperinsulinemia and abnormal glucose metabolism, clearly reproducing the pathological characteristics of T2DM.

IR and abnormal glucose metabolism are reported to be linked with ER stress (16, 24, 25), however, no research to date had explored whether ER stress was involved in the IR induced by SCH. Based on the important role of the liver in glucose metabolism and insulin action (26, 27), we chose it as the main organ to research. Consistent with our previous research, hepatic ER stress was triggered in SCH mice. We then rectified the ER stress response by using 4-PBA. Interestingly, IR status in SCH mice improved with alleviation of the ER stress level. Hence, it appears that ER stress played a crucial role in SCH-induced abnormal glucose metabolism and IR. Moreover, TSH may directly bind to hepatic TSHR and lead to increased ER stress. O-YuKwon et al. demonstrated that TSH could increased the expression of the endoplasmic reticulum resident 29 kDa protein (ERp29) in the FRTL-5 cells (18). TSH may have the similar effect on the activation of ER stress in hepatocytes. These findings may have implications for the primary prevention and treatment of individuals with diabetes.

The IRE1α/XBP-1 branch of the unfolded protein response (UPR) is one of the three classical pathways involved in ER stress (28). Upon initiation of ER stress, Bip dissociates from the stress sensor IRE1α leading to its activation by trans autophosphorylation which, in turn, catalyzes the excision of XBP-1 in the cytoplasm forming the activated mode of XBP-1 (XBP-1s) triggering UPR (29–31). Concurrently, activated IRE1α (p-IRE1α) stimulates the phosphorylation of JNK and IRS-1 suppressing the action of insulin, which results in IR. However, overexpression of XBP1s in murine embryonic fibroblast (MEF) cells suppressed ER-stress-induced JNK activation and increased activation by tyrosine phosphorylation of insulin-receptor substrate 1 (IRS-1) (16). PI 3-kinase (PI3K) is a crucial factor for insulin signaling (32), containing a regulatory subunit (p85α) and a catalytic subunit (p110α). There exists an interaction between p85α/p85β and XBP-1. In hepatocytes, p85α and p85β form heterodimers that dissociate upon insulin stimulation and interact with XBP1s to facilitate its nuclear translocation. Nuclear XBP1s then induces the expression of UPR target genes including chaperones and endoplasmic reticulum-associated degradation (ERAD) components, and improves insulin sensitivity (33–35). Therefore, ER stress can induce IR but, at the same time it can enhance expression of XBP-1 and promote the transcription of target genes which mitigate against IR. Although we observed the involvement of IRE1α/XBP-1 in our study, the specific role and mechanism of the IRE1α/XBP-1 pathway on IR in SCH still requires further research.

The strengths of the study may be that the relationship between SCH and diabetes were obtained by re-analyzing the data of a mature database (NHANES, 1999 ~ 2002). The findings are relatively credible and objective. However, due to the retrospective study design, causal relationships cannot be completely established. Then, we demonstrated the abnormal glucose metabolism and IR using the SCH mouse model and discovered the potential mechanism. Although the observations look strictly correlative, the exact mechanism still need to be further investigated.

In summary, our results demonstrated that ER stress, predominantly involving the IRE1α/XBP-1 pathway, may play a pivotal role in abnormal glucose metabolism and IR induced during SCH. Ameliorating ER stress can relieve IR in SCH which may suggest potential strategies for the prevention and treatment of diabetes.

## Methods

### Source and Screening of Respondents

The National Health and Nutrition Examination Survey (NHANES) is an epidemiological survey of the health of United States residents. Our data was sourced from the NHANES database from 1999 to 2002 which was downloaded from *https://doi.org/10.5061/dryad.d5h62* (14). The total number of subjects with TSH, T4 and GLU values was 2,152. Because SCH and diabetes are uncommon in younger individuals, we focused the analysis on subjects over 18 years, which produced a sample size of 1,534. We further excluded respondents with malignant tumors, overt hypothyroidism, subclinical, and clinical hyperthyroidism leading to a final dataset of 1,318 subjects. From this group, 54 cases were defined as SCH and the remaining 1,264 subjects were assigned as euthyroid controls. The screening process is shown in Supplementary Figure 1.

### Study Outcome Definition

We defined subjects suffering from SCH as those with a TSH range over 4.5 mU/L (TSH > 4.5 mU/L) and a normal T4 range from 4.5 to 13.2 μg/dL in accordance with previous research reports (36). A normal TSH range in serum was defined as from 0.4 to 4.5 mU/L (36). Diabetes mellitus was diagnosed by: 1. a past medical history according to the adult questionnaire and, 2. fasting plasma glucose > 7.0 mmol/L (125 mg/dL) (14). Abnormally high GLU was defined as over 6.0 mmol/L (108 mg/dL).

### Animals and Treatment

All mice were housed in an SPF room with controlled lighting (12 h on, 12 h off), and the temperature was maintained at 23°C.

### Generation of SCH Mice

Male C57BL/6 mice (7 weeks old) were obtained from Vital River Corporation (Beijing, China). All mice were fed and allowed to acclimatize for 1 week. The mice were then divided into two groups: one group (MMI-treated group, *n* = 6–8) was administered MMI (0.08 mg/kg·BW·d), a drug that inhibits thyroid hormone synthesis, in drinking water. The other group was provided with a corresponding volume of vehicle (control group, *n* = 6–8). The drinking water consumed was estimated using a 10-ml graduated cylinder every 3 days. We weighed the mice every 2 weeks. Water consumption and the MMI dose were adjusted every 3 days based on the last drinking water measurements and body weights. Following MMI was administration of MMI for 12 weeks the mice were fasted for 6 h and then euthanized using pentobarbital sodium. Serum was collected immediately prior to sacrificing the mice and serum samples were tested for FT3 (free triiodothyronine), FT4, and TSH in order to ensure the success of the SCH model.

### Oral Glucose Tolerance Test (OGTT) and Insulin Tolerance Test (ITT)

At weeks 13 and 14 of MMI administration in drinking water, we performed the OGTT) and ITT, as described previously, to evaluate glucose metabolism (36, 37). In brief, for the OGTT, mice were administered glucose (2 g/kg body weight) after a 12 h fast and the level of blood glucose were measured at 0, 30, 60, and 120 min using a glucometer. For the ITT, mice fasted for 5 h were injected intraperitoneally with insulin (1.0 U/kg body weight, human insulin, Lily), and blood glucose levels were determined at 0, 15, 30, 60, and 90 min after injection. The area under the curve (AUC) was calculated. Following MMI administration for 14 weeks, the mice were fasted for 6 h and then euthanized using pentobarbital sodium. Serum samples were collected immediately prior to sacrificing the mice, and tested for insulin and glucose concentrations.

### Treatment of Mice With 4-Phenylbutyrate

Following verification of the SCH state which was maintained for 2 weeks (i.e., MMI was used in drinking water for 14 weeks), SCH mice were intraperitoneally injected with 4-phenylbutyrate (4-PBA, P21005, Sigma-Aldrich, St. Louis, MO, USA) in phosphate-buffered saline (PBS) or with PBS only. Control mice (non-SCH) were simultaneously also injected with 4-PBA or with vehicle. 4-PBA was administered at a dose of 100 mg/kg·BW·d for 4 weeks. The specific protocols were described previously (13). During the 17th 18th weeks of MMI administration we performed the OGTT and the ITT, respectively, to evaluate glucose metabolism.

Following MMI administration for 18 weeks, mice were fasted for 6 h and were then euthanized using pentobarbital sodium. Serum and liver samples were collected and processed as described previously.

### Thyroid Function Determination

Serum FT3 and FT4 levels were determined by competitive radioimmunoassay (RIA) using an FT3 RIA Kit (Tianjin Jiuding, Tianjin, China) and an FT4 RIA Kit (Tianjin jiuding, Tianjin, China) by following the manufacturer's instructions. Serum TSH was determined using a mouse ELISA kit (MyBioSource, San Diego, California, USA) following the product manual.

### Blood Parameters

Serum insulin levels were assayed using an ELISA kit. Serum glucose level was determined using an Olympus AU5400 automatic biochemical analyzer (Olympus Co., Ltd., Tokyo, Japan) in Shandong Provincial Hospital. HOMA-IR was calculated as: fasting serum glucose (mmol/L) × fasting serum insulin (mIU/L)/22.5, is a fasting insulin resistance index, which is widely used as an indicator of pancreatic β cell function in clinics (37). ISI (insulin sensitivity index) was calculated as: 1/[fasting serum glucose (mmol/L) × fasting serum insulin (mIU/L)] (38) for the population-based study.

### Western Blot Analysis

Liver and cells were lysed in RIPA buffer with a protease inhibitor cocktail, PMSF, and sodium orthovanadate (Santa Cruz Biotechnology, Santa Cruz, CA). The protein concentration was quantified using a BCA protein assay (Pierce Biotechnology, Inc., Rockford, IL). Aliquots of prepared homogenates containing 80 μg of protein were resolved by SDS-PAGE, transferred to a membrane and blotted with specific antibodies: anti-phospho-IRE1α (ab48187), anti-IRE1α (ab37073) (Abcam, Cambridge, MA), anti-XBP1 (sc-8015) (Santa Cruz Biotechnology, CA), anti-phospho-eIF2α (#3398), anti-eIF2α (#5324) (Cell Signaling Technology, Boston, MA), and anti-Bip (No.11587-1-AP) (ProteinTech, Wuhan, China). The same membrane was stripped and re-blotted with anti-GAPDH antibody (Cwbiotech, Beijing, China) as a loading control.

### Statistical Analysis

Data were analyzed using SPSS 17.0. Data were expressed as the mean ± standard deviation (M ± SD), median (interquartile range) or percentage (%). Differences between the SCH patients and euthyroid controls were compared via the Mann-Whitney *U*-test or chi-square test. Logistic regression was used to analyze the risk for diabetes in different groups.

In the animal studies, differences between means were compared using either an unpaired Student's *t*-test for two-group comparisons or one-way analysis of variance (ANOVA) (Dunnett's *t*-test or LSD test) for multiple comparisons. All of the calculated *p*-values were two-sided, and *p*-values < 0.05 were considered to be statistically significant.

### Study Approval

Our research was approved by the Ethics Committee of Shandong Provincial Hospital (Jinan, China). The Shandong Provincial Hospital Animal Care and Use Committee approved the procedures for all animal experiments and the methods were performed according to the approved guidelines.

## Ethics Statement

Our research was approved by the Ethics Committee of Shandong Provincial Hospital (Jinan, China). The Shandong Provincial Hospital Animal Care and Use Committee approved the procedures for all animal experiments, and the methods were performed according to the approved guidelines.

## Author Contributions

The work presented here was carried out in collaboration between all authors. CX and LZ wrote the main manuscript text. LZ and KW performed the experiments. LG and CX conceived of the project and designed the experiments. YL and KW prepared all the animals. DJ and JX prepared data analysis and figures. All authors reviewed the manuscript.

### Conflict of Interest Statement

The authors declare that the research was conducted in the absence of any commercial or financial relationships that could be construed as a potential conflict of interest.

## Supplementary Material

Supplementary Figure 1 Study design and flow chart.

Supplementary Figure 2 Male C57/BL6 mice were given methimazole (MMI, 0.08 mg/kg·BW·d, SCH group) in drinking water or a corresponding volume of vehicle (control group) for 12 weeks. **(A)** The plasma TSH level was assayed at the 12th week (*n* = 6). **(B)** The plasma FT4 level was assayed at the 12th week (*n* = 6). The results are expressed as the mean ± SD.^*^*p* < 0.05 compared with control.

Supplementary Figure 3 The expression of JNK in SCH mice. Male C57/BL6 mice were given methimazole (MMI, 0.08 mg/kg·BW·d, SCH group) or a corresponding volume of vehicle (control group) for 14 weeks, the expression of JNK were detected by western blot (*n* = 4–6). ^*^*p* < 0.05 compared with control.

Supplementary Figure 4 We established SCH mouse model using MMI. When MMI was applied for 14 weeks, 4-PBA was given at a dose of 100 mg/kg·BW·d for 4 weeks, and mice were divided into four subgroups: vehicle treated control mice group (con + veh group), 4-PBA treated control mice group (con + 4-PBA group), vehicle treated SCH mice group (SCH + vehicle group), and 4-PBA treated SCH mice group (SCH + 4-PBA group). **(A)** The plasma TSH level was assayed at the 12th week (*n* = 4–6). **(B)** The plasma FT4 level was assayed at the 12th week (*n* = 4–6). The results are expressed as the mean ± SD.^*^*p* < 0.05 compared with control.
