# Supplementary figures and images for: Abnormal Glucose Metabolism and Insulin Resistance Are Induced via the IRE1α/XBP-1 Pathway in Subclinical Hypothyroidism
Source: Front Endocrinol (Lausanne). 2019 May 17;10:303. doi: 10.3389/fendo.2019.00303 (PMC6533547; doi:10.3389/fendo.2019.00303)

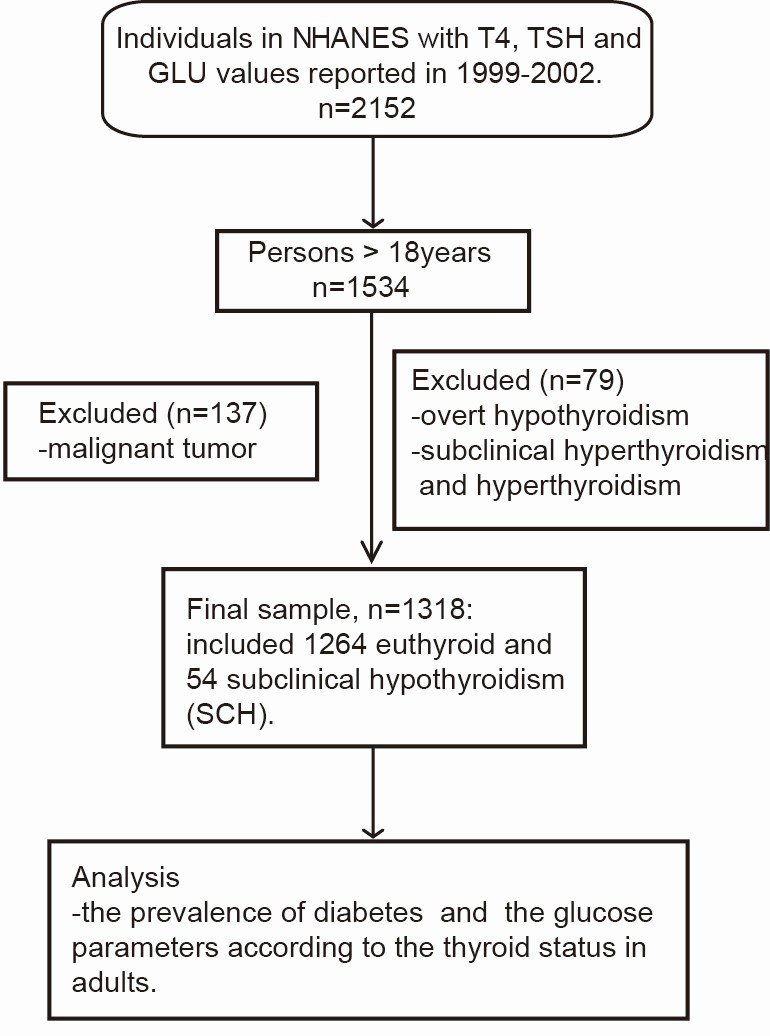

Supplement: Supplementary Figure 1 — Study design and flow chart. [file Image_1.jpg]

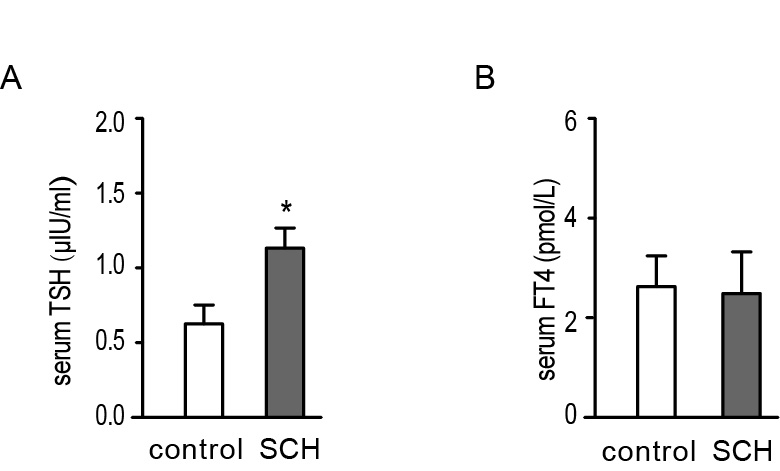

Supplement: Supplementary Figure 2 — Male C57/BL6 mice were given methimazole (MMI, 0.08 mg/kg·BW·d, SCH group) in drinking water or a corresponding volume of vehicle (control group) for 12 weeks. (A) The plasma TSH level was assayed at the 12th week (n = 6). (B) The plasma FT4 level was assayed at the 12th week (n = 6). The results are expressed as the mean ± SD.*p < 0.05 compared with control. [file Image_2.JPEG]

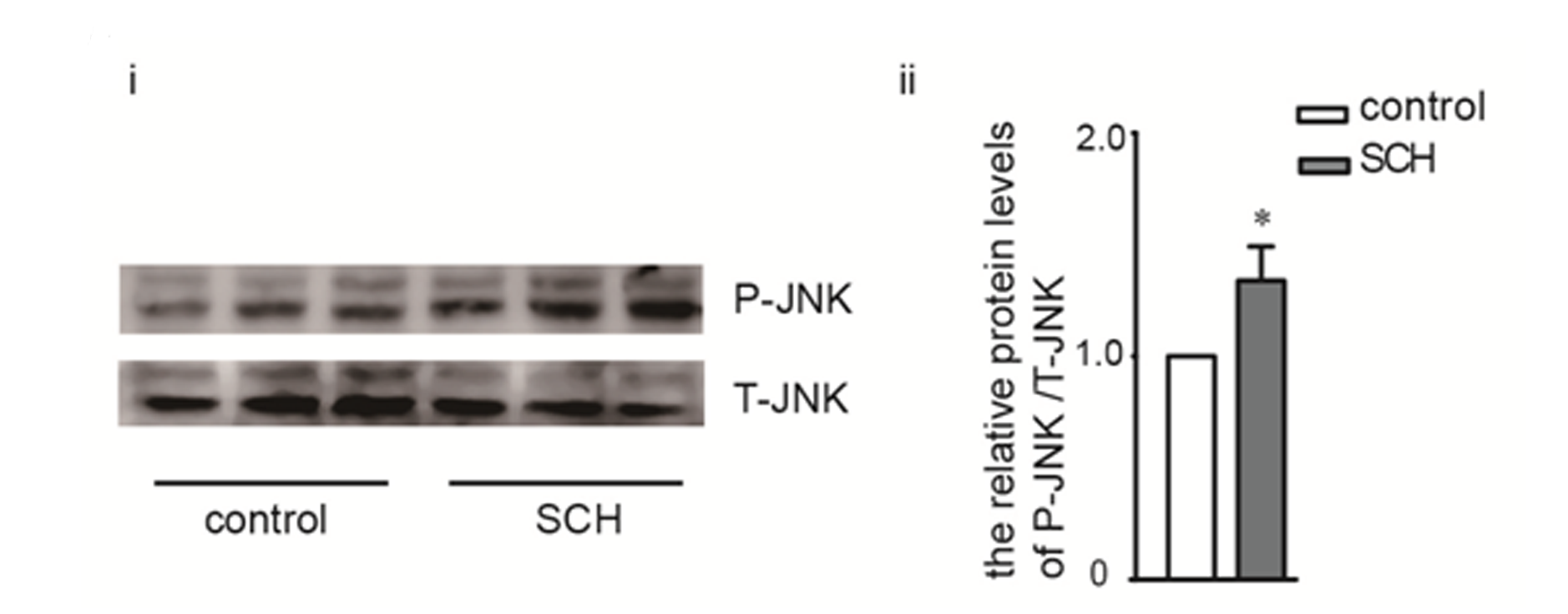

Supplement: Supplementary Figure 3 — The expression of JNK in SCH mice. Male C57/BL6 mice were given methimazole (MMI, 0.08 mg/kg·BW·d, SCH group) or a corresponding volume of vehicle (control group) for 14 weeks, the expression of JNK were detected by western blot (n = 4–6). *p < 0.05 compared with control. [file Image_3.tif]

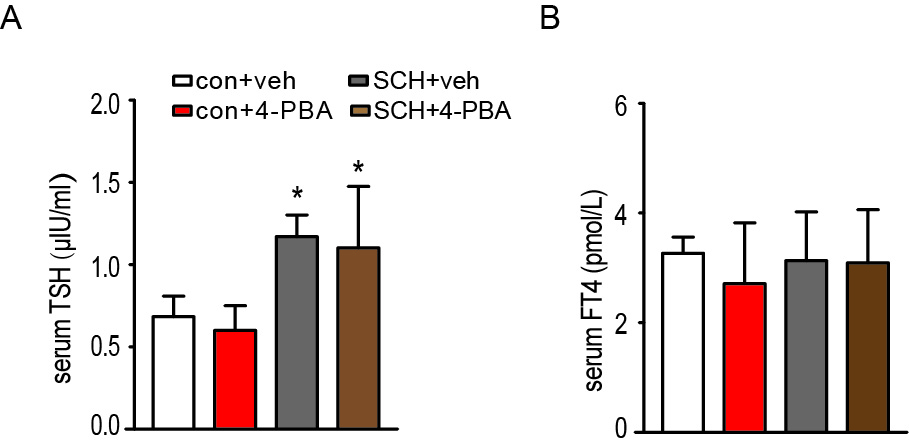

Supplement: Supplementary Figure 4 — We established SCH mouse model using MMI. When MMI was applied for 14 weeks, 4-PBA was given at a dose of 100 mg/kg·BW·d for 4 weeks, and mice were divided into four subgroups: vehicle treated control mice group (con + veh group), 4-PBA treated control mice group (con + 4-PBA group), vehicle treated SCH mice group (SCH + vehicle group), and 4-PBA treated SCH mice group (SCH + 4-PBA group). (A) The plasma TSH level was assayed at the 12th week (n = 4–6). (B) The plasma FT4 level was assayed at the 12th week (n = 4–6). The results are expressed as the mean ± SD.*p < 0.05 compared with control. [file Image_4.jpeg]
